# Socioeconomic inequalities in women’s access to health care: has Ecuadorian health reform been successful?

**DOI:** 10.1186/s12939-020-01294-1

**Published:** 2020-10-09

**Authors:** Edy Quizhpe, Miguel San Sebastian, Enrique Teran, Anni-Maria Pulkki-Brännström

**Affiliations:** 1 Colegio Ciencias de la Salud, Universidad San Francisco de Quito, USFQ, Quito, Ecuador; 2 Department of Epidemiology and Global Health, Umeå University, Umea, Sweden

**Keywords:** Health inequality, Health reform, Universal health coverage, Socioeconomic determinants, Ecuador

## Abstract

**Background:**

Over the last 12 years, Ecuador has implemented comprehensive health sector reform to ensure equitable access to health care services according to need. While there have been important achievements in terms of health care coverage, the effects of these reforms on socioeconomic inequalities in health care have not been analysed. The present study assesses whether the health care reforms implemented in the decade between 2007 and 2017 have contributed to reducing the socioeconomic inequalities in women’s health care access.

**Methods:**

The present study was based on two waves (2006 and 2014) of the Living Standards Measurement Survey conducted in Ecuador. Data from women of reproductive age (15 to 49 years) were analysed to evaluate health care coverage across three indicators: skilled birth attendance, cervical cancer screening, and the use of modern contraceptives. Absolute risk differences were calculated between the heath care indicators and the socioeconomic variables using binomial regression analysis for each time period. The Slope Index of Inequality (SII) was also calculated for each socioeconomic variable and period. A multiplicative interaction term between the socioeconomic variables and period was included to assess the changes in socioeconomic inequalities in health care over time.

**Results:**

Access to health care increased in the three studied outcomes during the health sector reform. Significant reductions in inequality in skilled birth attendance were observed in all socioeconomic variables except in the occupational class. Cervical cancer screening inequalities increased according to education and occupation, but decreased by wealth. Only a poorer education was observed for modern contraceptive use.

**Conclusions:**

While most socioeconomic inequalities in skilled birth attendance decreased during the reform period, this was not the case for inequalities in cervical cancer screening or the use of modern contraceptives. Further studies are needed to address the social determinants of these health inequalities.

## Background

Universal Health Coverage (UHC), defined as equitable access to health services with the aim of improving a population’s health, is proposed as a global policy by the United Nations [1]. The main components of UHC are (i) the delivery of quality essential health services according to need, and (ii) the financial protection of users from hardship, including possible impoverishment due to out-of-pocket payments [2]. Additionally, UHC means an investment in human capital to promote sustainable economic growth, development, and well-being [3].

To achieve UHC, a country is required to implement relevant changes and to delineate strategies based on human rights and equity within its political processes. Interventions including a more effective health system, increased decentralisation, wide and effective social engagement, and the strengthening of regulatory frameworks have been advocated [4].

Latin America in general provides an interesting regional case in terms of health care reforms because of the strong political commitments to UHC that have been made there since the ‘80s [5]. In several countries, the introduction of welfare reforms targeted to empower the poorest people (women in particular) via conditional cash transfer schemes to reduce poverty, have resulted in an increase in access and use of maternal and child health care services [6]. Similarly, increases in the level of public health care financing have contributed to expanding coverage in socially disadvantaged groups and reduced maternal and infant mortality. The experience of Brazil, Costa Rica, and Mexico has shown that providing financial risk protection can reduce catastrophic health expenditure amongst the poorest populations [4, 7]. This, and the potential lessons learnt from other countries who are aiming for UHC, was acknowledged internationally when, in 2015, *The Lancet* published a collection of papers charting the complex political, economic, and social forces that shaped health policy-making when Latin American countries tried to implement health system reforms [8]. These articles provided the most extensive review of the historical processes of several countries and their challenges over the previous 30 years [9]. An article by Wagstaff et al. published the same year (2015) assessed progress towards UHC using data from 112 household surveys from 20 Latin American countries between 1990 and 2013. The overall conclusion was that all countries were moving in the right direction, but were still far from achieving full UHC [10]. However, several studies examining the impact of health care reforms on equity in countries like Brazil, Mexico, and Colombia have suggested that the implemented reforms did not lead to improvements. This was due to internal and external factors such as huge fragmentation, poor delivery, and unsustainable financing [11–13].

Ecuador is an upper-middle income country, with high income inequality (Gini index 44.7 in 2017) and an ethnically diverse population. The majority are mestizos (a mixture between Spanish and indigenous people), but 28% belong to ethnic minorities, including indigenous, Afro-Ecuadorian descendants, and Montubios [14]. Between 1993 and 2006, Ecuador had eight different governments and experienced administrative instability, corruption, and social violence. At the same time, health care funding was slashed, and the government’s role in health care decreased significantly. In 2007, however, a new political proposal to reduce poverty and socioeconomic inequalities resulted in 10 years’ stable government. Comprehensive social and health reforms based on equity were introduced These involved ambitious changes to guarantee the right to health and UHC for all in Ecuador [15, 16].

During that decade, several relevant social indicators moved in a positive direction. Poverty levels dropped, employment and wages rose, the literacy rate increased, and income inequality diminished [17]. The Ministry of Health (MoH) introduced comprehensive health insurance policies to reduce cost sharing and fees, and all MoH health services gradually became free of charge. The share of public health expenditure in the gross domestic product increased from 2.2% in 2004 to 4.2% in 2015. This striking increase was mostly seen in medicines and supplies, vaccines, ambulances, new facilities, and health care equipment. New services such as home visits were introduced, particularly in rural areas and for vulnerable groups, and more than 4000 physicians were contracted. A legal framework of governance was also implemented to improve the relationship between the MoH, social insurance, and the private sector [18, 19]. Critics, however, have noted that the cost recovery mechanisms established between the public and private sector mainly benefited large private sector hospitals, and that the opportunity to establish a single health system was gradually fading [20]. During this period, social movements continued to call for a more egalitarian and participatory society [21].

Few studies have assessed the impact of the Ecuadorian health care reform on the population’s health. The literature has been mainly descriptive and focused on achievements in terms of coverage [19, 22]. A recent study has reported significant reductions in income inequalities in health care utilisation [23]. No-one, though, has paid attention to the potential impact of the reform on different socioeconomic groups, or specific health care outcomes. Monitoring and evaluating progress towards UHC is fundamental to improving health policy decisions and promoting equitable health systems [24]. Moreover, evaluations must incorporate all social subgroups, because national averages can mask health inequalities amongst the most disadvantaged.

The aim of this study was therefore to assess whether health care reform had any impact on progress towards UHC in Ecuador, and specifically, whether it has contributed to decreasing socioeconomic inequalities in three indicators of health care access amongst women.

## Method

### Study design

A cross-sectional study design was selected for this purpose. Secondary analysis based on publicly available data from the Living Standards Measurement Surveys 2006 and 2014 carried out by the National Institute of Statistics and Census (INEC) (*Instituto Nacional de Estadísticas y Censos*) was applied [17, 25]. A total of 55,000 households in 2006 and 109,000 in 2014 were selected. The sample selection was probabilistic in both surveys. It was stratified and proportional to the population size. The surveys for both periods comprised the same set of questions (on housing, ethnicity, education, economic activity, health care usage, and coverage).

A sub-sample of women of reproductive age (15 to 49 years) was selected from the total number of women recruited in 2006 (28,113) and in 2014 (55,397). The sample analysed included 13,781 women in 2006 and 26,767 women in 2014.

### Data collection

Primary sampling units were defined based on the grouping of census sectors in a first stage, and households in a second stage. The houses were randomly selected from a complete and updated list of occupied households and population by assigning the same selection probability to all houses [17]. Information was collected using face-to-face interviews by a group of properly trained pollsters from the same area and using a questionnaire designed to compile data from all members of the household. Data were gathered between November 2005 to October 2006 (before the reforms) and from November 2013 to October 2014 (during the reforms). The interviewers made as many visits as necessary to obtain information from the selected households.

### Dependent variables

Two indicators that represented promotion and prevention services were selected: cervical cancer screening and modern contraceptive use. A third indicator, skilled birth attendance, was selected to represent treatment coverage. These three outcomes have been used previously to indicate progress towards UHC, and were covered in the surveys [10, 26, 27].

Answers to the question “Who assisted you in your last delivery?” were used to capture skilled birth attendance (SBA). We defined assistance by a skilled professional if the answer was a physician, gynaecologist, nurse or obstetrician. Coverage of cervical cancer screening (CCS) was captured by the question “Have you ever had a Pap smear test?”, with yes or no options.

Modern contraceptive use (MCU) was assessed by asking two questions. “Are you using any contraceptive method?” was assessed via yes or no options. Those who responded positively were then asked, “What methods are you using to stop you getting pregnant?” Answers reporting female sterilisation (tubal ligation), implants, contraceptive injections, birth control pills, any type of intrauterine device, or condoms (female or male), were classified as modern contraceptives, while answers reporting other or a natural method were classified as non-modern methods.

### Socioeconomic variables

Place of residence was defined as living in either an urban or a rural area. Towns with less than 5000 inhabitants were considered rural. Ethnicity was based on self-identification. However, only two groups were used for the data analysis: non-indigenous people (including whites, mestizos, Afro-Ecuadorians, and Montubios) and indigenous people. This division was chosen because of the small sub-samples of some ethnic groups [28]. Education level was categorised as incomplete primary (comprising illiterate; literate but no formal education; and initial education categories), primary, secondary (middle secondary and technical) and higher education (undergraduate and postgraduate). We divided occupation into five categories and according to current occupation: managers, clerical support workers, farmers, plant and machine operators, and elementary occupations based on the international occupational classification [29]. A wealth index was generated using principal component analysis (PCA) based on household characteristics and assets (household entrance paving, roof, wall and floor material, type of house, cooking facility, cooking fuel, type of toilet, water source, lighting source, land line telephone, home internet, satellite TV, and household waste disposal). PCA was run separately for 2006 and 2014, and for each period it was divided into quintiles, with the first representing the richest.

### Data analysis

The population characteristics were summarised using descriptive statistics to calculate the prevalence of each variable in 2006 and 2014. Statistical differences between the two periods in the three health outcomes were determined using chi-square tests. Regression analysis was used to calculate absolute risk differences (ARD) between the heath care indicators and the socioeconomic variables. First, a binomial regression analysis for each of the periods was applied, and the Slope Index of Inequality (SII) was calculated to measure the extent of socioeconomic difference. The SII is a weighted measure of inequality that represents the absolute difference in the estimated values of a health indicator between the most advantaged and the most disadvantaged, while taking into consideration the size of all the other subgroups [1, 30]. Ridit scores, corresponding to the average cumulative proportion of the categories of each socioeconomic indicator, were created. As previously recommended [31], SII coefficients were obtained by generalised linear models, using identity link functions, with the outcome regressed on the ridit scores, separately by each socioeconomic indicator. The index is the slope of the resulting regression line, and represents the absolute difference expressed as percentage points between the fitted value of the outcome in the most and least advantaged on the socioeconomic scale. If there is no inequality, SII takes the value zero. Greater absolute values indicate higher levels of inequality. In this study, positive values indicate higher coverage in the advantaged subgroups and negative values indicate higher coverage in the disadvantaged subgroups. Finally, a multiplicative interaction term between the socioeconomic variables’ ridit scores and time period was included to assess the changes in socioeconomic inequalities in health care over time. Sample weights were applied to all analyses and 95% confidence intervals for significance were calculated. All analyses were conducted using Stata 15.1 statistical software.

## Results

### Population characteristics

Table 1 shows the characteristics of the study population in the two periods. One third lived in rural areas and the proportion who identified as indigenous was the same in both years. Nearly 40% had a primary education or less in 2006; an increase in the proportion with the lowest level of education was observed in 2014 compared with 2006. One-fifth of participants belonged to the lowest occupational class (elementary occupations) in 2006, and fewer clerical support workers but more farmers and elementary occupations were represented in 2014.

**Table 1:** Socioeconomic characteristics of Ecuadorian women of reproductive age in 2006 and 2014^a^

	2006		2014	
Variable	n	(%)	n	(%)
**Residence**				
Urban	9355	(67.88)	18,888	(70.56)
Rural	4426	(32.12)	7879	(29.44)
**Ethnicity**				
Whites/mestizos/afro	12,869	(93.38)	24,863	(92.88)
Indigenous	912	(6.62)	1904	(7.12)
**Education**				
Higher (highest)	2959	(21.47)	5596	(20.91)
Secondary	5560	(40.34)	11,995	(44.81)
Primary	4392	(31.87)	6200	(23.16)
Incomplete primary (lowest)	870	(6.31)	2976	(11.12)
**Occupational class**				
Managers (highest)	763	(13.95)	2730	(16.52)
Clerical support workers	2699	(49.36)	6085	(36.81)
Farmers	198	(3.63)	1677	(10.15)
Plants and machine operators	671	(12.28)	1392	(8.42)
Elementary occupations (lowest)	1136	(20.78)	4646	(28.10)
**Household wealth index**				
1st quintile (richest)	2971	(21.69)	8325	(31.26)
2nd quintile	2950	(21.54)	6170	(23.17)
3rd quintile	2958	(21.59)	5221	(19.60)
4th quintile	2485	(18.14)	3945	(14.81)
5th quintile (poorest)	2334	(17.04)	2974	(11.17)

^a^Sample weights were applied to calculate the prevalence

The coverage of all three health care outcomes increased significantly over time. The proportion of women attended by a skilled professional during childbirth increased from 85.6% in 2006 to 93.7% in 2014 (*p*-value < 0.01); the coverage of cervical cancer screening from 51.3 to 59.8% (*p*-value < 0.01); and the coverage of modern contraceptive use from 40.7 to 48.4% (*p*-value < 0.01)*.*

### Socioeconomic inequalities in health

Table 2 presents the absolute risk differences and SII for skilled birth attendance. The SII was statistically significantly positive in 2006 for residence, ethnicity, education, and wealth, indicating higher coverage in the socially advantaged subgroups; however, the SII was not statistically significant for occupational class. Between 2006 and 2014, statistically significant reductions in socioeconomic inequalities were observed in terms of place of residence (SII difference = − 28.11; 95% CI: − 32.69 to − 23.53), ethnicity (SII difference = − 27.41; 95% CI: − 38.05 to − 16.78), education (SII difference = − 20.04; 95% CI: − 22.49 to − 17.59) and wealth (SII difference = − 3.96; 95% CI: − 4.47 to − 3.45). In contrast, there was a significant but small increase in inequality by occupational class (SII difference = 3.53; 95% CI: 2.75 to 4.31).

**Table 2:** Prevalence of coverage, absolute risk differences and slope index of inequality for skilled birth attendance

	Prevalence		Absolute risk difference (ARD)		Slope index of inequality (SII) difference
	2006	2014	2006	2014	(2006–2014)
**Residence**					
Urban	95.96	98.39	Ref	Ref	
Rural	67.46	83.94	28.50 (26.37,30.62)	14.44 (13.19,15.69)	
SII			57.00 (52.75,61.25)	28.88 (26.38,31.39)	- 28.11 (− 32.69,- 23.53)
**Ethnicity**					
Whites/mestizos/afro	89.79	96.79	Ref	Ref	
Indigenous	37.87	58.58	51.91 (47.40,56.42)	38.20 (34.92, 41.49)	
SII			103.83 (94.81,112.85)	76.41 (69.85,82.98)	- 27.41 (− 38.05, − 16.78)
**Education**					
Higher (highest)	99.37	99.61	Ref	Ref	
Secondary	94.77	96.95	4.60 (3.56,5.64)	2.65 (2.09,3.20)	
Primary	73.03	85.62	26.34 (24.40,28.28)	13.98 (12.68,15.29)	
Incomplete primary (lowest)	53.25	88.62	46.12 (39.64,52.60)	10.98 (8.90,13.06)	
SII			36.06 (34.06,38.06)	16.09 (14.74,17.29)	- 20.04 (− 22.49, − 17.59)
**Occupational class**					
Managers (highest)	92.08	99.53	Ref	Ref	
Clerical support workers	92.13	98.03	−0.04 (3.63,3.55)	1.49 (0.78,2.21)	
Farmers	98.61	76.08	- 6.52 (− 10.64, − 2.41)	23.45 (20.57,26.32)	
Plants and machine operators	95.67	94.90	- 3.58 (− 7.73,0.56)	4.63 (2.76,6.49)	
Elementary occupations (lowest)	89.01	85.73	3.07 (− 1.30,7.44)	13.80 (12.17,15.43)	
SII			0.41 (− 0.44,1.26)	3.94 (3.59,4.30)	3.53 (2.75,4.31)
**Household wealth**					
1st quintile (highest)	99.49	99.39	Ref	Ref	
2nd quintile	97.08	98.30	2.40 (1.31,3.49)	1.09 (0.49,1.68)	
3rd quintile	92.62	95.52	6.86 (5.32,8.41)	3.86 (2.98,4.75)	
4th quintile	82.09	90.22	17.39 (15.12,19.66)	9.17 (7.77,10.58)	
5th quintile (lowest)	61.89	78.19	37.60 (34.87,40.32)	21.20 (19.11,23.29)	
SII			7.39 (6.91,7.87)	3.43 (3.16,3.71)	- 3.96 (− 4.47, − 3.45)

The results for the cervical cancer screening inequalities are presented in Table 3. The SII for cervical cancer screening was also statistically significantly positive for residence, ethnicity, education, and wealth in 2006, but not significant for occupational class. No significant differences by residence (SII difference = − 1.96; 95% CI: − 4.05, 0.13) or ethnicity (SII difference = − 0.86; 95% CI: − 4.48, 2.75) were observed between the two periods. Significant increases in inequality by education (SII difference = 6.69; 95% CI: 3.15, 10.22) and occupation class (SII difference = 3.37; 95% CI: 2.45, 4.28) were observed. The only significant observed reduction was in inequality by wealth (SII difference = − 1.76; 95% CI: − 2.47, − 1.06).

**Table 3:** Prevalence of coverage, absolute risk differences and slope index of inequality for cervical cancer screening

	Prevalence		Absolute risk difference (ARD)		Slope index of inequality (SII) difference
	2006	2014	2006	2014	(2006–2014)
**Residence**					
Urban	55.53	63.13	Ref	Ref	
Rural	42.33	51.89	13.20 (11.43,14.97)	11.23 (9.93,12.53)	
SII			26.40 (22.86,29.94)	22.47 (19.87,25.07)	- 3.92 (− 8.11, 0.26)
**Ethnicity**					
Whites/mestizos/afro	52.93	61.53	Ref	Ref	
Indigenous	28.17	37.63	24.76 (21.72,27.80)	23.89 (21.64,26.15)	
SII			49.52 (43.44,55.61)	47.79 (43.28,52.31)	- 1.73 (− 8.97,5.51)
**Education**					
Higher (highest)	56.34	68.09	Ref	Ref	
Secondary	52.28	58.85	4.05 (1.83, 6.27)	9.24 (7.73,10.74)	
Primary	51.99	71.66	4.35 (2.03,6.66)	- 3.56 (− 5.22, − 1.91)	
Incomplete primary (lowest)	24.26	23.57	32.08 (28.71,35.44)	44.51 (42.56,46.47)	
SII			16.84 (13.75,19.93)	23.53 (21.43,25.64)	6.69 (3.15, 10.22)
**Occupational class**					
Managers (highest)	56.92	80.36	Ref	Ref	
Clerical support workers	60.58	69.78	- 3.65 (− 7.62, − 0.31)	10.58 (8.69,12.46)	
Farmers	56.41	65.93	0.51 (− 7.23,8.25)	14.43 (11.72,17.14)	
Plants and machine operators	67.78	72.29	- 10.85 (− 15.84, −5.87)	8.07 (5.29,10.85)	
Elementary occupations (lowest)	55.40	61.75	1.52 (− 3.02,6.07)	18.60 (16.56,20.65)	
SII			0.22 (− 0.70,1.15)	3.59 (3.12,4.06)	3.37 (2.45, 4.28)
**Household wealth**					
1st quintile (highest)	58.79	63.14	Ref	Ref	
2nd quintile	56.06	64.63	2.72 (0.21,5.24)	- 1.48 (− 3.06,0.09)	
3rd quintile	52.29	59.14	6.49 (3.97,9.02)	3.99 (2.30,5.68)	
4th quintile	48.44	55.51	10.35 (7.70,12.99)	7.63 (5.76,9.49)	
5th quintile (lowest)	37.23	47.66	21.55 (18.91,24.20)	15.48 (13.40,17.55)	
SII			5.27 (4.66,5.87)	3.50 (3.07,3.93)	- 1.76 (− 2.47, −1.05)

Patterns of social inequalities in the use of modern contraceptives for family planning were more diverse (Table 4). In 2006, the socially advantaged groups were not more prevalent except in terms of ethnicity. Between 2006 and 2014, a non-significant change in inequality by residence was observed, where coverage was higher among the rural population in 2014 (SII Difference = 3.45; 95% CI: − 7.99, 10.78). No significant reductions in inequality by ethnicity were observed (SII difference = − 1.53; 95% CI: - 10.50, 7.43). Significant reductions in terms of education (SII difference = 6.91; 95% CI: 3.10, 10.72) were observed over time. Inequalities by occupation class (SII difference = − 1.88; 95% CI: − 2.87, − 0.08) and wealth (SII difference = − 1.79; 95% CI: − 2.55, − 1.02), had small significant increases.

**Table 4:** Prevalence of coverage, absolute risk differences and slope index of inequality for modern contraceptives use

	Prevalence		Absolute risk difference (ARD)		Slope index of inequality (SII) difference
	2006	2014	2006	2014	(2006–2014)
**Residence**					
Urban	40.07	47.45	Ref	Ref	
Rural	42.07	51.17	- 1.99 (− 3.90, 0.08)	- 3.72 (− 0.51,- 2.31)	
SII			- 3.99 (− 7.80,- 0.01)	- 7.44 (− 10.27,- 4.62)	3.45 (− 7.99,10.78)
**Ethnicity**					
Whites/mestizos/afro	40.98	48.86	Ref	Ref	
Indigenous	34.17	42.82	6.81 (2.84,10.78)	6.04 (3.42,8.65)	
SII			13.62 (5.68,21.56)	12.08 (6.85,17.31)	- 1.53 (− 10.50,7.43)
**Education**					
Higher (highest)	33.25	42.97	Ref	Ref	
Secondary	39.94	47.32	−6.69 (−8.87, −4.50)	- 4.35 (−5.99, − 2.71)	
Primary	49.20	62.57	−15.94 (−18.30,13.58)	- 19.60 (− 2.14, − 17.74)	
Incomplete primary (lowest)	28.74	32.71	4.50 (0.48,8.51)	10.26 (7.90, 12.61)	
SII			- 15.84 (−19.07, 12.62)	- 8.93 (− 11.28, − 6.58)	6.91 (3.10,10.72)
**Occupational class**					
Managers (highest)	42.91	43.43	Ref	Ref	
Clerical support workers	44.78	50.59	- 1.87 (− 5.98,2.24)	- 7.15 (− 9.48, − 4.83)	
Farmers	29.97	61.23	12.93 (5.48,20.38)	- 17.80 (− 20.96, − 14.64)	
Plants and machine operators	36.23	56.41	6.67 (1.49,11.86)	- 12.97 (− 16.32, − 9.63)	
Elementary occupations (lowest)	44.89	50.79	- 1.97 (− 6.70,2.74)	- 7.36 (− 9.81, − 4.90)	
SII			0.49 (− 0.47, 1.47)	- 1.38 (− 1.91,- 0.84)	- 1.88 (− 2.87,- 0.08)
**Household wealth**					
1st quintile (highest)	36.56	41.09	Ref	Ref	
2nd quintile	39.43	49.14	- 2.86 (− 5.42, − 0.31)	- 8.04 (− 9.75, − 6.33)	
3rd quintile	41.31	51.70	- 4.74 (− 7.31, − 2.17)	- 10.60 (− 12.41, − 8.79)	
4th quintile	43.93	53.50	- 7.36 (− 10.10, − 4.62)	- 12.40 (− 14.40, − 10.40)	
5th quintile (lowest)	44.04	56.94	- 7.48 (− 10.38, − 4.57)	- 15.84 (− 18.11, − 13.58)	
SII			- 2.14 (− 2.79, − 1.48)	- 3.93 (− 4.40, − 3.46)	- 1.79 (− 2.55,-1.02)

## Discussion

The present study assessed the socioeconomic inequalities in Ecuadorian women’s health care access in the context of comprehensive social reforms based on equity, and primary health care oriented health sector reform. The results show that during the period 2006 to 2014 access to health care increased and health inequalities between certain social groups decreased. Despite this, some social inequalities in health care have remained or have even increased over time.

### Skilled birth attendance

Several factors could explain the moderate increase in coverage (from an already high level) observed in skilled birth attendance; these include the rise in the number of health care facilities with maternity services, the expansion of the health workforce (particularly into rural areas), the thorough implementation of the free maternity programme (*Ley de Maternidad Gratuita*) that has been in place since 2005, and the increase in enrolment on the national health insurance scheme amongst public employees and farmers, which includes free maternal and child care [19, 24, 32].

Large reductions in inequalities were observed for rural, indigenous, and the lowest education groups, though inequalities remained high in 2014. To improve intercultural health care, the MoH incorporated guidelines for traditional practices in all governmental health care services in 2008 [33]; however, several national studies have demonstrated inconsistent levels of integration of traditional practices during pregnancy and childbirth [34, 35]. Similarly, several barriers have been observed in access to health services amongst indigenous women [36], and research has shown that this same group tend to be less aware of obstetric warning signs, as well as the use of health services, than mestizas in the country [37].

Studies from Latin America have demonstrated that the integration of traditional birth attendants (TBAs) within the formal health system increases skilled birth attendance and the use of sexual and reproductive health services [38, 39]. However, this strategy was abandoned during the reform period [40], although public health policies to improve articulation between TBAs and the formal health system were recently announced [41].

### Cervical cancer screening

Cervical cancer is the third cause of death in women in Ecuador [42]. However, the proportion of women screened for cervical cancer was low in all socioeconomic groups in both periods. As with skilled birth attendance, high inequalities were observed in relation to place of residence, ethnicity and education; conversely, there was little reduction in inequality from 2006 to 2014.

A pap test (cytology) is the basis for cervical cancer screening, and is provided free of charge in all public health care facilities. Testing is promoted when women use health care services. Although access to health facilities improved significantly over time, a weak application of health promotion policies and persisting barriers to screening might explain both the low coverage and inequalities [43]. Latin American studies have identified a number of obstacles, including feelings of shame, negative perceptions of health workers, concern about the test results and procedures, and previous negative experiences [44, 45]. Low education, poverty, lack of access to health insurance, and limited use of health services have also been reported as barriers to screening in countries in the Americas region [46–50]. Similarly, high ethnic disparities in Ecuador have been observed between indigenous and mestizo women regarding preventative knowledge about breast and cervical cancer and sexually transmitted infections [51].

Comprehensive cancer management has traditionally been one of the weakest public health strategies in the country, with extreme fragmentation between the preventive and curative components of the health system. In an attempt to strengthen this area, the MoH developed a national strategy for cancer care in 2017 to ensure equitable access along the care continuum [52]. This will hopefully contribute to increased access and decreased inequalities in the future.

### Modern contraceptive use

The coverage of modern contraceptive use increased from 40.7 to 48.4%, which is lower than the average coverage reported in the Americas region as a whole (68%) [53]. The increase is modest in relation to the huge investment in the purchase and supply of modern contraceptives in primary care and access to female sterilisation (ligation) at the secondary level of care, especially after childbirth. In 2013, the MoH issued new guidelines to guarantee the availability of family planning methods and the promotion of sexual and reproductive health at primary care level nationally [54], which hopefully will have contributed to an increase in coverage more recently.

The socioeconomic inequalities in coverage for both periods were surprisingly concentrated in disadvantaged groups, except amongst indigenous women. A study of rural Ecuadorian women has shown how they have moved from biomedical to traditional care in accessing family planning due to the inconsistent availability of contraceptive methods in public health services [55]. Similarly, programmes that do not respond to community needs or lack cultural sensitivity have impeded access even when contraceptives are widely available [56–58].

Studies have also demonstrated how bureaucratic barriers in free choice contexts can limit the use of health services, while the attitudes and behaviours of maternal health care providers in interactions with clients can discourage the use of contraceptives [59]. There are, however, positive experiences in the country that have overcome some of these difficulties. A recent study of women from low resource communities in Ecuador showed how increasing economic opportunities, preventing gender-based violence, and valuing their community role contributed to empowerment in the use of contraceptive methods [60].

### Methodological considerations

The strengths of the present study include its large population-based random sample and the national representation of different socioeconomic groups. The possibility of selection bias was thus precluded. The application of the same questionnaires in the two periods that were studied and the inclusion of several socioeconomic variables are also strong features.

Given that this was a population-based study, some questionnaire answers may have been subject to response and recall bias. Although the institution responsible for conducting the surveys carried out rigorous interviewer training, the extent of this is difficult to determine. While changes in socioeconomic inequalities in health have herein been attributed to health reforms, other factors may have played a part, so any inferences from the results should be treated with caution. Finally, though the period of health reform assessed in the study was 2007–2017, the available surveys were from 2004 (pre-reform) and 2014 (during). It is possible that the results may have been different by the end of the decade. This will be possible to assess when the next national survey becomes available.

## Conclusions

Overall, the measures that have been taken to achieve UHC have been successful. The reforms in Ecuador have led to improved access to health care services, but the examined health indicators show a limited reduction in inequalities. Most of the socioeconomic inequalities in terms of skilled birth attendance decreased, but only small decreases were observed in cervical cancer screening and modern contraceptive use. Several interventions would be needed to address the persistence of health inequalities for indigenous and rural women, such as cultural competency training for health workers and the implementation of intercultural health policies at the primary health care level. These would require the involvement of indigenous organisations. Government efforts are also needed to influence positively the social determinants of health inequalities.

## Data Availability

Data used in this study are publicly available and can be retrieved from https://www.ecuadorencifras.gob.ec/institucional/home/.

## Abbreviations

UHC: Universal Health Coverage
LAC: Latin America and the Caribbean
MoH: Ministry of Health
INEC: Instituto Nacional de Estadísticas y Censos
SBA: Skilled Birth Attendance
CCS: Cervical Cancer Screening
MCU: Modern Contraceptive Use
PCA: Principal Component Analysis
ARD: Absolute Risk Differences
SII: Slope Index of Inequality

## References

[1] World Health Organization (2013). Handbook on health inequality monitoring: with a special focus on low- and middle-income countries.

[2] Universal Health Coverage. http://www.who.int/universal_health_coverage/en/. Accessed on Dec 2019.

[3] World Health Organization (2008). CSDH, closing the gap in a generation: health equity through action on the social determinants of health.

[4] Atun R, de Andrade L, Almeida G, Cotlear D (2015). Dmytraczenko et al. health-system reform and universal health coverage in Latin America. Lancet..

[5] Dmytraczenko T, Almeida G (2015). Toward universal health coverage and equity in Latin America and the Caribbean.

[6] Cecchini S, Madariaga A (2011). Conditional cash transfer programmes: the recent experience in Latin America and the Caribbean.

[7] Barreto ML, Rasella D, Machado DB, Aquino R, Lima D (2014). Monitoring and evaluating progress towards universal health coverage in Brazil. PLoS Med.

[8] Horton R, Das P (2015). Universal health coverage: not why, what, or when—but how?. Lancet.

[9] Atun R, de Andrade LO, Almeida G, Cotlear D, Dmytraczenko T, Frenz P, Garcia P, Gómez-Dantés O, Knaul FM, Muntaner C, de Paula JB, Rígoli F, Serrate PC, Wagstaff A (2015). Health-system reform and universal health coverage in Latin America. Lancet.

[10] Wagstaff A, Dmytraczenko T, Almeida G, Buisman L (2015). Assessing Latin America’s progress toward achieving universal health coverage. Health Aff.

[11] Esteves RJ (2012). The quest for equity in Latin America: a comparative analysis of the health care reforms in Brazil and Colombia. Int J Equity Health.

[12] Giovanella L, Mendoza-Ruiz A (2018). de Carvalho Amand Pilar a, Cantanhede da Rosa M, Branco martins G, Soares Santos I, et al. universal health system and universal health coverage: assumptions and strategies. Cien Saude Colet.

[13] Frenk J, Gomez-Dantes O (2018). Health systems in Latin America: the search for universal health coverage. Arch Med Res.

[14] Censo de Población y Vivienda 2010 Ecuador. https://www.ecuadorencifras.gob.ec/base-de-datos-censo-de-poblacion-y-vivienda/. Accessed on December 2019.

[15] Asamblea Constituyente (2008). Constitución de la República del Ecuador.

[16] Plan Nacional del Buen Vivir 2009–2013. https://www.planificacion.gob.ec/wpcontent/uploads/downloads/2012/07/Plan_Nacional_para_el_Buen_Vivir_(version_resumida_en_espanol).pdf. Accessed on Dec 2019.

[17] Living Standard Measures Survey 2014. http://www.ecuadorencifras.gob.ec/documentos/web-inec/ECV/ECV_2015/. Accessed on Dec 2019.

[18] De Paepe P, Echeverría Tapia R, Aguilar SE (2012). Ecuador's silent health reform. Int J Health Serv.

[19] Espinosa V, Acuña C, De la Torre D, Tambini G (2017). La reforma en salud del Ecuador. Rev Panam Salud Publica.

[20] Iturralde P (2015). Privatización de la salud en Ecuador Estudio de la interacción pública con clínicas y hospitales privados.

[21] Friederic K, Burke B (2019). La “revolución ciudadana” and social medicine: undermining community in the state provision of health care in Ecuador. Glob Public Health.

[22] Malo M, Malo N (2014). Reforma de salud en Ecuador: nunca más el derecho a la salud como un privilegio. Rev Peru De Med Exp Salud Publica.

[23] Granda M, Jimenez W (2019). The evolution of socioeconomic health inequalities in Ecuador during a public health system reform (2006–2014). Int J Equity Health.

[24] Boerma T, Eozenou P, Evans D, Evans T, Kieny M-P (2014). Monitoring progress towards universal health coverage at country and global levels. PLoS Med.

[25] Living Standard Measures Survey 2006. http://www.ecuadorencifras.gob.ec/condiciones-de-vida-ecv-bases-de-datos/. Accessed on Dec 2019.

[26] Boerma T, AbouZahr C, Evans D, Evans T (2014). Monitoring intervention coverage in the context of universal health coverage. PLoS Med.

[27] Organización Mundial de la Salud y Banco Mundial (2014). Monitoreo del progreso hacia la cobertura universal de salud a nivel nacional y global. Marco de trabajo, medidas y metas.

[28] Sistema de Indicadores Sociales del Ecuador 2015. http://www.siise.gob.ec/siiseweb/. Accessed on Dec 2019.

[29] INEC (2010). Manual de usuario CIUO 08-Clasificación Internacional de Ocupaciones 2008.

[30] Regidor E (2004). Measures of health inequalities: part 2. J Epidemiol Community Health.

[31] Khang Y-H, Yun S-C, Lynch JW (2008). Monitoring trends in socioeconomic health inequalities: it matters how you measure. BMC Public Health.

[32] Lucio R, Villacrés N, Henríquez R (2011). Sistema de salud de Ecuador [the health system of Ecuador]. Salud Publica Mex.

[33] Ministerio de Salud Pública (2008). Guía Técnica para la atención del parto culturalmente adecuado.

[34] Gallegos C, Watersb W, Sebert KA (2017). Discourse versus practice: are traditional practices and beliefs in pregnancy and childbirth included or excluded in the Ecuadorian health care system?. Int Health.

[35] Llamas A, Mayhew S (2018). “Five hundred years of medicine gone to waste”? Negotiating the implementation of an intercultural health policy in the Ecuadorian Andes. BMC Public Health.

[36] Waters W, Ehlers J, Ortega F, Sebert KA (2018). Physically demanding labor and health among indigenous women in the Ecuadorian highlands. J Community Health.

[37] Bustamante G, Mantilla B, Cabrera-Barona P, Barragan E, Soria S (2019). Awareness of obstetric warning signs in Ecuador: a cross-sectional study. Public Health.

[38] Byrne A, Morgan A (2011). How the integration of traditional birth attendants with formal health systems can increase skilled birth attendance?. Int J Gynaecol Obstet.

[39] Martinez C (2018). Discriminación y colonialidad en el Ecuador de Rafael Correa (2007–2017). Alteridades.

[40] Lalander R, Ospina P (2012). The indigenous movement and citizen revolution in Ecuador. Cuestiones Políticas.

[41] Ministerio de Salud Pública (2016). Articulación de prácticas y saberes de las parteras ancestrales en el Sistema Nacional de Salud, Manual.

[42] Nacimientos y Defunciones 2015. https://www.ecuadorencifras.gob.ec/estadisticas-de-nacimientos-y-defunciones-2015/. Accessed on Dec 2019.

[43] Goldberg D (2012). Social justice, health inequalities and methodological individualism in US health promotion. Public Health Ethics.

[44] Agurto I, Bishop A, Sanchez G, Betancourt Z, Robles S (2004). Perceived barriers and benefits to cervical cancer screening in Latin America. Prev Med.

[45] Luffy S, Evans D, Rochat R (2015). “Siempre me critican”: barriers to reproductive health in Ocotal, Nicaragua. Rev Panam Salud Publica.

[46] Soneji S, Fukui N (2013). Socioeconomic determinants of cervical cancer screening in Latin America. Rev Panam Salud Publica.

[47] Murillo R, Almonte M, Pereira A, Ferrer E, Gamboa O (2008). José Jerónimo, Lazcano-Ponce E. cervical cancer screening programs in Latin America and the Caribbean. Vaccine.

[48] Bermedo-Carrasco S, Peña-Sanchez J, Lepnurm R, Szafron M, Waldner C (2015). Inequities in cervical cancer screening among Colombian women: a multilevel analysis of a nationwide survey. Cancer Epidemiol.

[49] Abiodun OA, Olu-Abiodun OO, Sotunsa JO, Oluwole FA (2014). Impact of health education intervention on knowledge and perception of cervical cancer and cervical screening uptake among adult women in rural communities in Nigeria. BMC Public Health.

[50] Islam R, Billah B, Hossain N, Oldroyd J (2017). Barriers to cervical cancer and breast cancer screening uptake in low-income and middle-income countries: a systematic review. Asian Pac J Cancer Prev.

[51] Castillo R. Ethnic disparities in the reproductive and sexual health screening practices of Ecuadorian women. MsC thesis. University of Texas. Open Access Theses & Dissertations; 2015.

[52] Ministerio de Salud Pública (2017). Estrategia nacional para la atención integral del cáncer en el Ecuador.

[53] Pan American Health Organization (2016). Health situation in the Americas: Core indicators.

[54] Ministerio de Salud Pública (2013). Reglamento para regular el acceso de métodos anticonceptivos.

[55] Arnold J, Flint J, Casapulla S, Nieto C, Grijalva M (2019). Medical pluralism in maternal health-seeking behavior of rural women in southern Ecuador. Health Care Women Int.

[56] Wurtz H (2012). Indigenous women of Latin America: unintended pregnancy, unsafe abortion, and reproductive health outcomes. Pimatisiwin.

[57] Westgard C, Ally Rogers A, Bello G, Rivadeneira N (2019). Health service utilization, perspectives, and health-seeking behavior for maternal and child health services in the Amazon of Peru, a mixed-methods study. Int J Equity Health.

[58] Dansereau E, Schaefer A, Hernandez B, Nelson J, Palmisano E (2017). Perceptions of and barriers to family planning services in the poorest regions of Chiapas, Mexico: a qualitative study of men, women, and adolescents. Reprod Health.

[59] Goicolea I (2001). Exploring women's needs in an Amazon region of Ecuador. Reprod Health Matters.

[60] Feld H, Rojas V, Linares AM (2019). “We keep quiet”: exploring the context of pregnancy intention in a low-resource community in Ecuador. Sex Reprod Health Matters.

